# Full-length transcriptome-referenced analysis reveals crucial roles of hormone and wounding during induction of aerial bulbils in lily

**DOI:** 10.1186/s12870-022-03801-8

**Published:** 2022-08-27

**Authors:** Jingrui Li, Meiyu Sun, Hui Li, Zhengyi Ling, Di Wang, Jinzheng Zhang, Lei Shi

**Affiliations:** Key Laboratory of Plant Resources and China National Botanical Garden, Institute of Botany, Chinese Academy of Sciences, Beijing, Xiangshan 100093 China

**Keywords:** Bulbil induction, Full-length transcriptome, Oriental hybrid lily cultivar ‘Sorbonne’, Hormone- and wounding-regulated network

## Abstract

Aerial bulbils are important vegetative reproductive organs in *Lilium*. They are often perpetually dormant in most *Lilium* species, and little is known about the induction of these vegetative structures. The world-famous Oriental hybrid lily cultivar ‘Sorbonne’, which blooms naturally devoid of aerial bulbils, is known for its lovely appearance and sweet fragrance. We found that decapitation stimulated the outgrowth of aerial bulbils at lower stems (LSs) and then application of low and high concentrations of IAA promoted aerial bulbils emergence around the wound at upper stems (USs) of ‘Sorbonne’. However, the genetic basis of aerial bulbil induction is still unclear. Herein, ‘Sorbonne’ transcriptome has been sequenced for the first time using the combination of third-generation long-read and next-generation short-read technology. A total of 46,557 high-quality non-redundant full-length transcripts were generated. Transcriptomic profiling was performed on seven tissues and stems with treatments of decapitation and application of low and high concentrations of IAA, respectively. Functional annotation of 1918 DEGs within stem samples of different treatments showed that hormone signaling, sugar metabolism and wound-induced genes were crucial to bulbils outgrowth. The expression pattern of auxin-, shoot branching hormone-, plant defense hormone- and wound-inducing-related genes indicated their crucial roles in bulbil induction. Then we established five hormone- and wounding-regulated co-expression modules and identified some candidate transcriptional factors, such as MYB, bZIP, and bHLH, that may function in inducing bulbils. High connectivity was observed among hormone signaling genes, wound-induced genes, and some transcriptional factors, suggesting wound- and hormone-invoked signals exhibit extensive cross-talk and regulate bulbil initiation-associated genes via multilayered regulatory cascades. We propose that the induction of aerial bulbils at LSs after decapitation can be explained as the release of apical dominance. In contrast, the induction of aerial bulbils at the cut surface of USs after IAA application occurs via a process similar to callus formation. This study provides abundant candidate genes that will deepen our understanding of the regulation of bulbil outgrowth, paving the way for further molecular breeding of lily.

**Supplementary Information:**

The online version contains supplementary material available at 10.1186/s12870-022-03801-8.

## Introduction

Lily is a perennial geophyte and cultivated for its flower and bulb. They are usually clonally propagated using daughter bulbs, bulb scales, and aerial bulbils [1, 2]. Aerial bulbils originate from the meristems of lily leaf axils, and each bulbiferous plant can produce dozens or even hundreds of bulbils. Mature bulbils can be easily separated from the mother plant and directly planted into the soil, giving rise to new independent plants. For bulbiferous species, including *L. lancifolium*, *L. sulphureum*, *L. sargentiae*, and *L. bulbiferum*, bulbil reproduction is the best choice as it is labor-saving, convenient, and efficient. However, most lily species (∼110 species) do not generate bulbils naturally. Since bulbils are numerous and propagate very easily, it is beneficial to learn how to induce the formation of bulbils in those species that do not produce these propagules [1]. Recently, we observed that decapitation and IAA application could induce the generation of bulbils in *Lilium* Oriental hybrid ‘Sorbonne’ [3]. ‘Sorbonne’ is a widely cultivated variety with dramatic market demand due to its favorable blooms, sweet fragrance, and strong resistance. A comprehensive description underlying the mechanisms of bulbil induction in those species that do not produce these propagules is challenged, but it is required for lily reproduction.

The formation of bulbils is a complex developmental process that is regulated by genetic and environmental factors. Only a few plant species, such as *Agave tequilana*, *Dioscorea batatas*, *Allium sativum*, *Titanotrichum oldhamii*, *L. sulphureum* and *Pinellia ternata* rely on bulbils for reproduction [4]. After disrupted apical dominance, axillary bulbils quickly form in *T. oldhamii* [5] and *A. tequilana* [6], while exogenous auxin restores apical dominance and inhibits bulbil formation. Additionally, only a few genes have been shown to participate in the regulation of bulbil formation, such as *GFLO* in *T. oldhamii* [7], *AtqKNOX1/2* and *AtqMADS1/2/4/6/7* in *A. tequilana* [8, 9], and *carotenoid cleavage dioxygenase 8* (*CCD8*) in *Solanum tuberosum* [10]. The previous studies of the development of bulblets and bulbils in lily [4, 11] have been limited to short-read sequencing technology and many transcripts are fragmented. Lily’s enormous genome size (about 36 Gb) is an obstacle for genomic information mining. With the emergence of full-length transcriptome sequencing technology, gene function studies in species without reference genomes have become more convenient [12–17]. Furthermore, the short reads produced by Illumina sequencing have resulted in lower error rates than PacBio sequencing [12, 17]. Therefore, hybrid sequencing strategies are both more affordable and of higher quality than single sequencing alone.

Previous histological studies of lily have indicated that aerial bulbils are derived from axillary meristems [4]. Much of our current knowledge of the control of axillary branch growth centers on the roles of plant hormones, particularly auxin, cytokinin and strigolactones (SLs). Two comprehensive models have been developed, involving an auxin transport canalization-based concept and a second messenger theory [18]. In the first model, the continual flow of auxin from the apical buds is thought to prevent auxin flow from the axillary bud, resulting in excess auxin in axillary buds, causing them to remain dormant. In the later model, cytokinin and SL are regarded as the second messenger of auxin that can directly move into axillary buds, whereas auxin suppresses cytokinin production or promotes the expression of SL biosynthesis genes. Recent discoveries revealed that SLs and gibberellin (GA) might act antagonistically in control of shoot branching [19, 20], and abscisic acid (ABA) signaling may play a much more prominent role in the initiation of axillary bud outgrowth [21]. On the other hand, another study postulated that sugars, rather than auxin, are necessary and sufficient to regulate the very earliest periods of bud outgrowth following decapitation [22].

Studies of organ regeneration have indicated that wounding activates a very fast regeneration response, and reactivation of cell proliferation at wound sites can lead to the formation of a cell mass called callus and the subsequent establishment of shoot or root apical meristems [23–26]. Wound stress alone, however, is often insufficient to provoke the entire suite of regenerative responses. Plant hormones are also essential forthe lateral root or shoots formation. The plant hormones auxin and cytokinin are well established as efficient inducers of callus in tissue culture. The Arabidopsis explants usually induce callus first by incubating on an auxin-rich medium and subsequently induce shoot on a cytokinin-rich medium [27]. A recent study showed that a dynamic jasmonate acid (JA) wave cooperates with histone methylation to upregulate a pulse of auxin production and promote de novo root regeneration in response to wounding [28].

Our study showed that decapitation stimulated the outgrowth of aerial bulbils at lower stems (LSs), and then application of low and high concentrations of IAA promoted aerial bulbils emergence around the wound at upper stems (USs) of ‘Sorbonne’. Here, we report the first full-length transcriptome reference sequences from ‘Sorbonne’ by using the PacBio long-read Iso-Seq technique. A total of 46,557 high-quality non-redundant full-length transcripts were generated. The expression pattern of auxin-, shoot branching hormone-, plant defense hormone- and wound-inducing-related genes indicated their crucial roles in the induction of bulbils. By establishing hormone- and wounding-regulated gene co-expression modules, we identified key genes underlying the initiation of bulbils in lily. Wound stress and hormonal cues may integrate and collectively regulate a large collection of genes implicated in bulbil developmental decisions at USs and LSs.

## Results

### Induction of bulbils and the phytohormone changes in lily

**Fig. 1:**
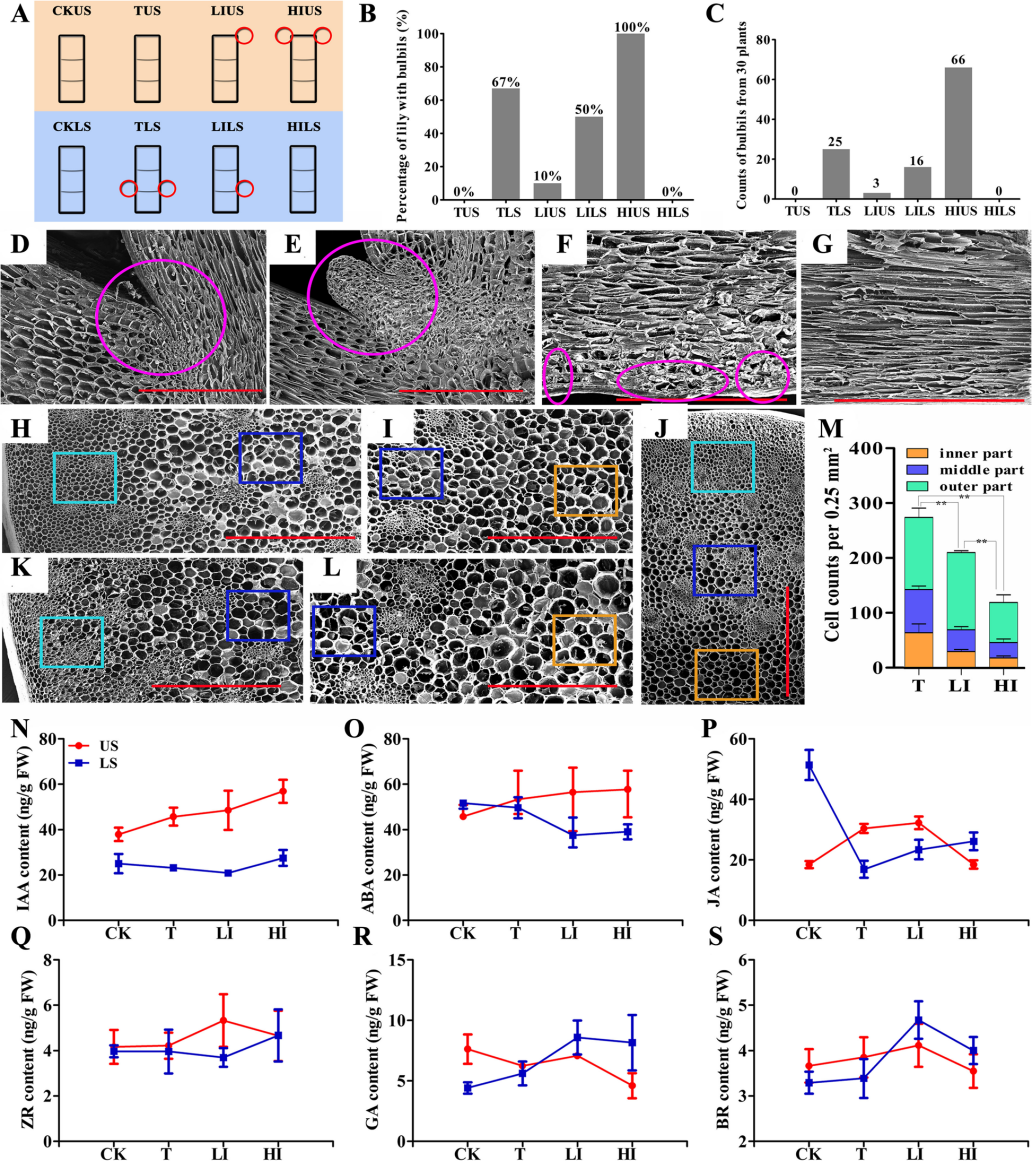
Effects of decapitation and auxin on bulbils of lily. **A** Schematic plot showed the growth of bulbils after decapitation and application of auxin at USs and LSs of lily. Squares in orange and blue background indicate USs and LSs, respectively. Red circles represent bulbils. **B**, **C** The percentage of lily with bulbils (**B**) and total bulbils number (**C**) of 30 lily plants was counted. **D**-**L**, Scanning electron microscopic images of USs and leaf axil. Scale bars = 1 mm (**D**-**L**). The leaf axils indicated with magenta circles of CK (**D**) and treated plants with bulbils (**E**). The vertical section of USs treated with HI (**F**) and CK (**G**). The callus-like cells were circled with magenta ovals (**F**). Cross-section of USs treated with T (**J**), LI (**H**, **I**) and HI (**K**, **L**). **M** Number of parenchymal cell from pith to epidermis (divided into inner, middle and outer part) was counted. The orange, blue and turquoise boxes were exampled for cell counts of inner, middle and outer parts of stem. Asterisks indicate significant differences of the total cell number among TUS, LIUS, and HIUS, as determined by the Tukey’s HSD test (** *P* < 0.01). **N-S** Changes in the concents of six endogenous hormones at USs and LSs of different treatments: IAAN (N), ZR (**O**), ABA (**P**), GA3 (**Q**), JA (**R**) and BR (**S**)

Decapitation stimulated the bulbils outgrowth, and IAA prevented this process at lower stems (LSs) of Oriental hybrid lily cultivar ‘Sorbonne’ in our previous study. Intriguingly, the application of IAA promoted bulbil emergence around the wound of upper stems (USs) after decapitation (Fig. 1A). LSs and USs of four types, including control plants (CK) and decapitated plants with free IAA (T), low concentration IAA (LI) and high concentration IAA (HI), were employed in the experiment, each with 30 plants. At LSs, a total of 25 bulbils were found in 20 decapitated plants, 16 bulbils emerged in 15 plants treated with LI, and no bud outgrowth in all plants treated with HI. At USs, there were 0, 3 and 66 bulbils generated in nodes of plants treated with T, LI and HI (Fig. 1B, C). Moreover, scanning electron microscopic (SEM) images of USs showed that parenchymal cells (from the inner part to outer part of the stem) of decapitated plants were small and serried.

In contrast, cell size was significantly larger, and the cell number was decreased in a dose-dependent manner when the decapitated lily was exposed to IAA (Fig. 1D-M). After 2 weeks of treatments, we observed that the LSs were burlier, and the leaves were greener and larger in treated plants than CK (Additional file 1: Fig. S1A-H). On the underground part of decapitated plants, the bulbs were larger and less senescent than the intact plants afterward (Additional file 1: Fig. S1J,K). Correspondingly, the bulblets, which developed from underground axillary buds, of decapitated plants were fatter (the average fresh weight of one bulblet produced by decapitated plants was 6.0-fold to control plants) as compared with CK (Additional file 1: Fig. S1I,L). Next, we measured six primary endogenous phytohormones [IAA, GA3, brassinosteroid (BR), zeatin-riboside (ZR), JA and ABA] of USs and LSs, both before and after treatment, using ELISA (Fig. 1N-S). It is not surprising that IAA concentration was elevated at USs and LSs after auxin treatment. In addition, ZR concentration was increased after IAA application at USs, but decapitation and LI application displayed a weak effect on ZR concentration at LSs. The GA3 and ABA concentration changes were opposite in an auxin-dose-dependent manner. JA concentration was strongly influenced by decapitation at both the LSs and USs in accordance with its defense function, while HI suppressed the induction at USs.

### Reconstruction of the full-length transcriptome of lily

**Fig. 2:**
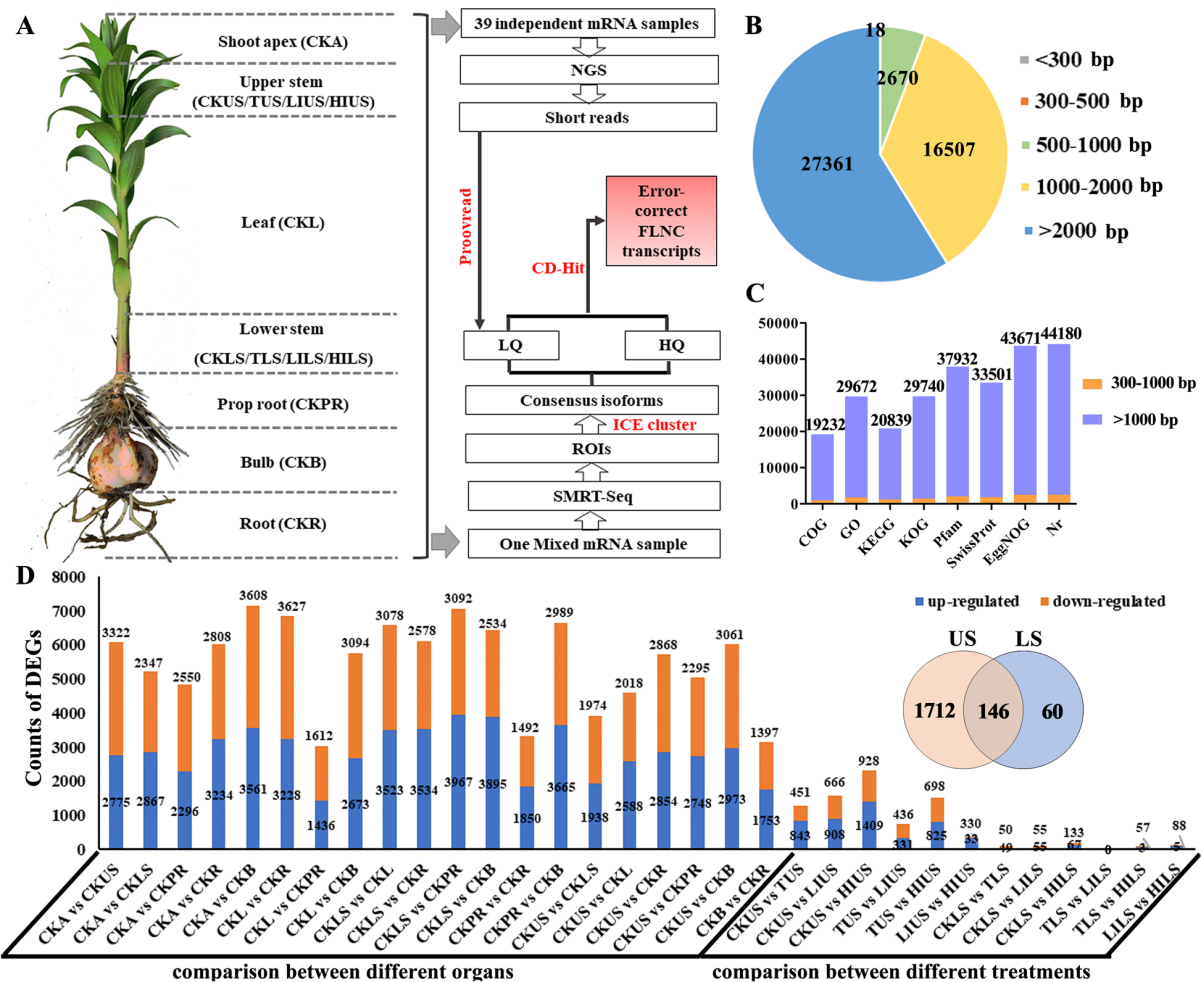
Reconstruction of the full-length transcriptome of lily without a reference. **A** The sequenced lily samples lily and experimental workflow. Seven organs (CKA, CKL, CKPR, CKR, CKB, CKUS and CKLS) and six stem samples with auxin-related treatment (USs and LSs of T, LI and HI) were used. Both SMRT- and RNA-Seq libraries were constructed and software names are in red. **B** Length distribution of transcripts produced from PacBio platform. **C** Annotation of full-length transcripts based on eight databases. **D** The number of up- and down-regulated genes in comparison between seven types of lily organ and upper stem or lower stem with or without IAA treatment. Overlapping of differentially expressed genes compared in pairs of all USs and LSs are shown

To investigate the molecular regulation of bulbil induction in lily, we first adopted a combination of NGS and SMRT-Seq technology to obtain a comprehensive transcriptome (Fig. 2A). A pooled RNA derived from various lily organs (see method) at the leaf expansion stage was subjected to SMRT sequencing using a PacBio Iso-Seq platform with seven cells (Additional file 2: Table S1). After filtering, a total of 5,736,140 subreads were generated; we identified 401,875 reads of insert (ROIs) with mean read quality of insert over 0.87 for each library (Additional file 1: Fig. S2A). Of all the ROIs, 175,230 were found to be full-length non-chimeric (FLNC) reads because each contained a 5′ primer, a 3′ primer, and a poly (A) tail. As expected, the average length of FLNC reads from the different libraries (1–2, 2–3, 3–5, and 5–8 kb) produced by SMRT-Seq were 1302, 2187, 3422, and 5551 bases, respectively (Fig. S2B). A total of 58,939 consensus isoforms were obtained using Iterative Clustering for Error Correction (ICE) (Additional file 1: Fig. S2C, D), in which we identified 43,342 high-quality isoforms (HQ) (accuracy > 99%) after using the Quiver algorithm combined with non-full length reads. We made further corrections to the rest of the low-quality isoforms (LQ) by utilizing Illumina HiSeq 2000 transcriptomic data from different stem samples (Additional file 2: Table S2). In total, 46,557 non-redundant transcripts with N50 at 3452 bp were obtained, and redundant sequences were trimmed to eliminate consensus isoforms using CD-HIT software. The length of majority (94.23%) FLNC transcripts were over 1000 bp, and short transcripts (< 500 bp) accounted for only 0.04% (Fig. 2B). A total of 44,588 transcripts were annotated by alignment with seven databases, including 19,232 (43.1%), 29,672 (66.5%), 20,839 (46.7%), 29,740 (66.7%), 37,932 (85.1%), 33,501 (75.1%), 43,671 (98.0%) and 44,180 (99.1%) genes were annotated to the COG, GO, KEGG, KOG, Pfam, Swiss-Prot, EggNOG and Nr databases, respectively. Only 5.7% of annotated genes had less than 1000 bp (Fig. 2C). We also tried to de novo assemble the Illumina clean reads from 39 libraries with Trinity (v2.4.0), resulting in 196,289 unigenes with N50 at 912 bp. Comparative analysis of transcripts produced by Pacbio long reads and Illumina assembled reads revealed that the number of complete CDs (44,965) is assembled by only Illumina reads was far less than Pacbio FlNC transcripts (75,819), as well as the transcript length (Additional file 1: Fig. S3). These results demonstrated that the combination of SMRT- and RNA-Seq provided a large number of high-quality transcripts for reference, which was of great importance for genomic studies on the lily.

### Differentially expressed genes of lily

Using full-length of lily transcriptome as a reference, Illumina clean reads of all 39 samples were aligned to them with acceptable mapping ratios between 58.35 and 73.27% (average 67.77%) (Additional file 2: Table S2). In each pairwise comparison, we summarized the up-or down-regulated genes among seven untreated organ groups and upper stem or lower stem groups with or without IAA treatment (Fig. 2D). Multiple genes are expressed differently across vegetative organs. The number of differentially expressed genes (DEGs) (Fold change> 2 and FDR < 0.05) ranged from 3048 (CKL vs CKPR) to 7169 (CKA vs CKB) across the pairwise comparison between different organs (Fig. 2D). In all, 9999 DEGs were detected in pairwise comparisons among seven organ groups and IAA-treated upper stems or lower stems groups and expression profile of all DEGs is shown in (Additional file 3: Data S1). After filtering low expression (FPKM < 0.1) and correlated transcripts (*p*-value threshold was 1e^− 3^), the overall 4347 transcripts were clustered into 11 optimized modules by conducting a weighted correlation network analysis (WGCNA) (Additional file 1: Fig. S4A; Additional file 4: Data S2). The expression pattern of extensive DEGs was in an organ-specific manner (Additional file 1: Fig. S4B).

To understand the effect of decapitation and auxin to bulbil outgrowth, pairwise comparisons were performed on four US sample sets (CKUS, TUS, LIUS, HIUS) and four LS sample sets (CKLS, TLS, LILS, HILS). Filtering genes with low expression levels and non-obvious variation trends between samples, we yielded a total of 1918 DEGs with 146 DEGs shared by US and LS sample sets (Fig. 2D). The DEGs specific to US and LS or shared by both US and LS were enriched in the carbohydrate metabolism pathway (Additional file 1: Fig. S5-S7), which may contain key regulators during bulbils induction.

### Genes in response to decapitation and auxin

#### Auxin-related genes

**Fig. 3:**
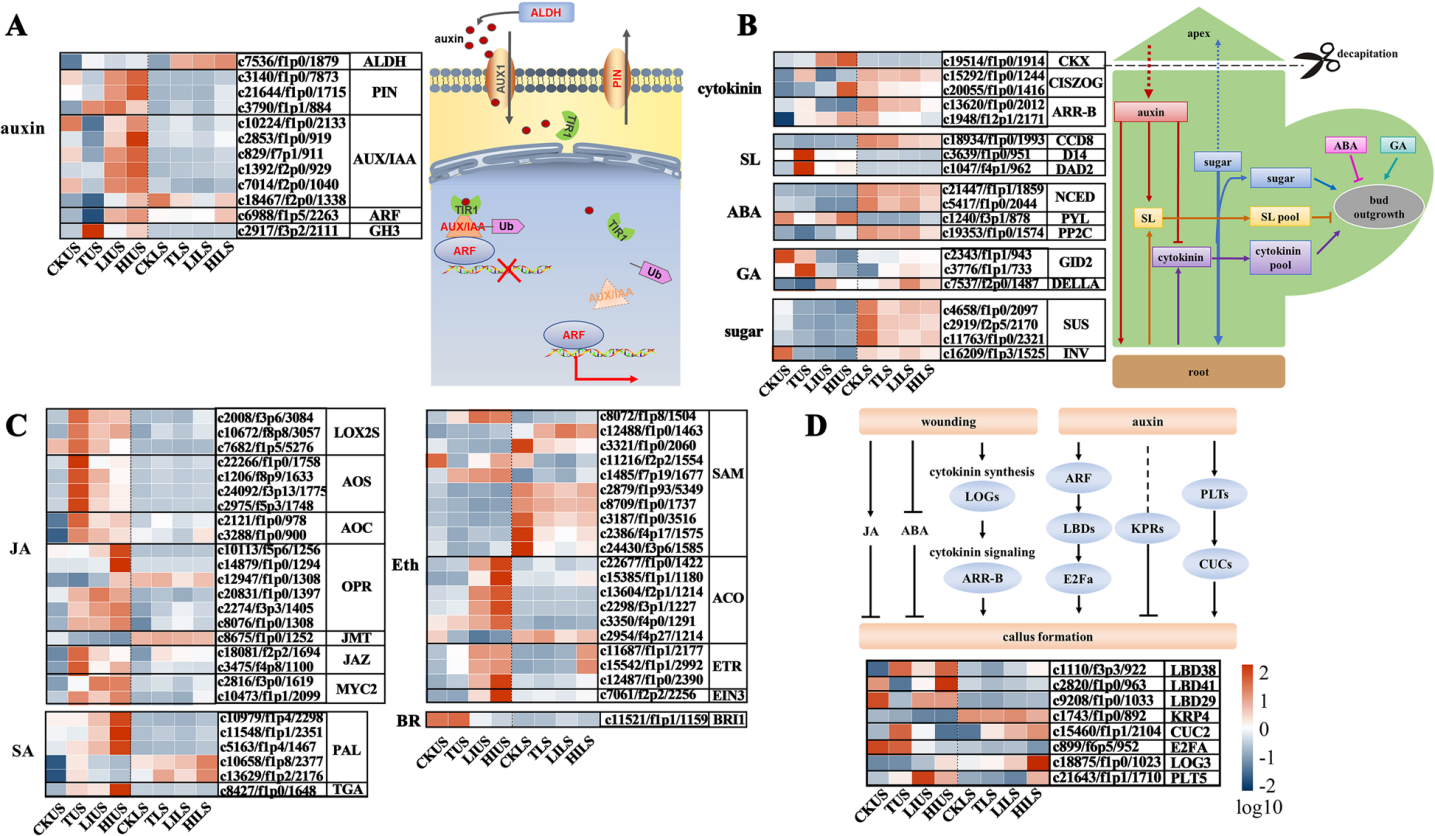
Heatmap depicting the expression profile of phytohormone and wound-related genes. **A** Expression atlas of genes related to auxin biosynthesis, transport and signal transduction. **B** Expression atlas of genes related to cytokinin, SL, GA and ABA biosynthesis and signaling pathway. **C** Expression atlas of genes related to JA, Eth, SA and BR biosynthesis and signaling pathway. **D** Expression atlas of wound-related genes

To elucidate the potential molecular mechanism by which IAA affects bulbil outgrowth in lily, the expression of genes involved in IAA biosynthesis, transport, and signal transduction was investigated. The expression level of gene *aldehyde dehydrogenase* (*ALDH*) and *polar transport capacities* (*PIN*) involved in auxin synthesis and transport, and *auxin/indole acetic acid* (*AUX/IAA*), auxin response factor (ARF), and *GH3* involved in auxin signal transduction significantly increased after treatment both in USs and LSs (Fig. 3A). Previous studies reported that the expression of *polar transport capacities 1* (*PIN1*) was correlated with primordium development [29, 30]. At USs, HI stimulated the expression of *PINs*, while LI displayed a weak inducement of these genes (Fig. 3A). Moreover, we found that the intensity of gene response to auxin signaling (*AUX/IAA* and *ARF*) was attenuated by decapitation but recovered after IAA application. The effects of treatments on LSs were weak, but a similar fluctuation trend was observed when compared to USs (Fig. 3A). TUS showed a considerable up-regulation of *GH3* genes, which encode indole-3-acetic acid-amino synthetase, that conjugates IAA to amino acids and then reduces IAA concentrations. Validation of *ARF* (c6988/f1p5/2263), *AUX/IAA* (c10224/f1p0/2133) and *PIN* (c3140/f1p0/7873) via qRT-PCR further confirmed our observations (Additional file 1: Fig. S8).

#### Shoot branching-related genes

We also report transcriptomic results in response to other four hormones (cytokinin, SL, GA, and ABA), which play roles in branching besides auxin [31–33]. These four hormones were derived from dimethylallyl pyrophosphate, isopentenyl pyrophosphate and geranyl pyrophosphate, which are produced by the methylerythritol 4-phosphate and mevalonate pathways. The interaction of four hormones may attribute to their common precursors. *CKX*, which related to the degradation of cytokinin was strongly induced after IAA application (LIUS, HIUS and HILS). Maybe the IAA application suppresses the cytokinin wave. The expression of the SLs synthetic gene, *CCD8*, was repressed by removing the shoot apex. The high expression of *decreased apical dominance 2* (*DAD2*) and *dwarf 14* (*D14*), which is involved in SL signaling, may be responsible for the repression of bulbil formation at USs in decapitated plants. For ABA, expression of *NCED* genes was down-regulated in IAA-treated plants. *Serine/ threonine protein phosphatases type 2C* (*PP2C*) showed a significant increase in response to IAA. For GA, the *GID2* was down-regulated after the IAA application, while *DELLA* showed the opposite trend (Fig. 3B). Sugars also govern bud release locally, as the *SUS* and *INV* genes were down-regulated following decapitation (Fig. 3B).

#### Plant defense-related genes

Ethylene, JA, and SA, play important roles in plant defense [31–33]. Most genes involved in JA biosynthesis, such as *LOX2S*, *AOS*, *AOC*, *OPR*, and signaling pathway, the JA repressor *jasmonate ZIM-domain protein* (*JAZ*) and *MYC2*, were significantly up-regulated by decapitation at USs. As for ethylene signaling response, the expression of *ACO*, *ethylene receptor* (*ETR*), and *EIN* were increased at LIUS and HIUS samples, and SAM was down-regulated at TLS, LILS, and HILS samples. The *brassinosteroid insensitive 1* (*BRI1*) were down-regulated after the application of IAA at US. The *TGA* and *PAL* involved in SA signaling were up-regulated at Uss by IAA exposure, leading to the activation of plant defense pathways (Fig. 3C).

#### Wound-induced genes

In this study, we observed callus-like cells close to the stem epidermis as well as many adventitious roots below the cut surface after treatment with HI but not after treatment with LI (Additional file 1: Fig. S1). Indeed, plants develop unorganized cell masses like callus and tumors in response to various biotic and abiotic stimuli. Our RNA-seq data showed that wounding causes dramatic changes in gene expression profiles, such as many genes related to JA biosynthesis and signaling, which was often induced by wounding, were up-regulated after decapitation treatment (Fig. 3C). *Cup-shaped cotyledon 2* (*CUC2*), which is important for shoot regeneration after wounding scales in lily, was primarily induced by decapitation and IAA treatments at USs. The expression of *lateral organ boundary domain* (*LBD*), *plethora* (*PLT*), and *lonely guy* (*LOG*), that act as critical nodes controlling callus formation and developmental decisions [25, 26], are strongly induced at USs after decapitation with IAA application (Fig. 3D).

### Identification of potential regulators of bulbil outgrowth using modular organization analysis

**Fig. 4:**
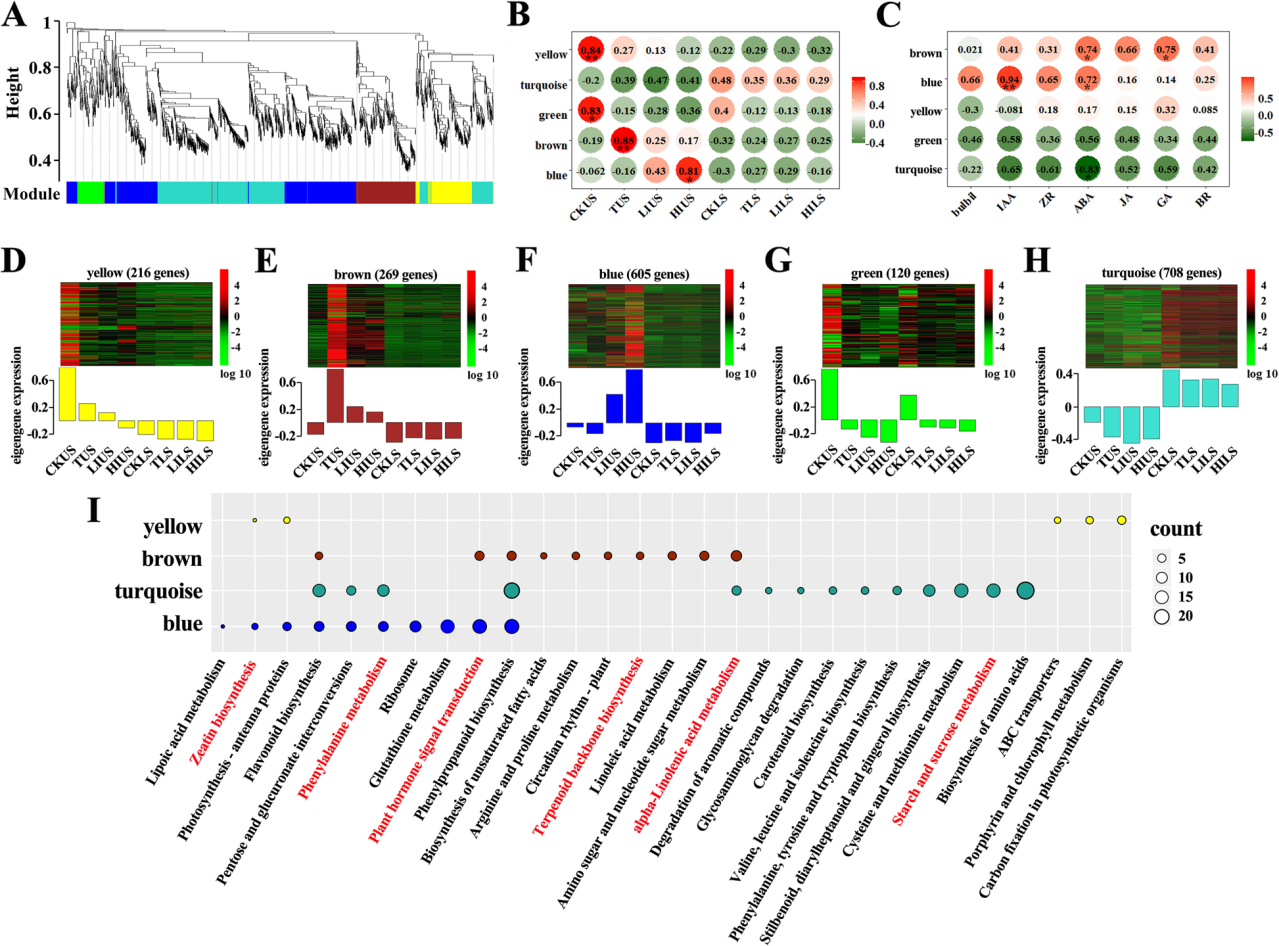
Weighted gene co-expression network of stem samples. **A** Hierarchical cluster tree showing coexpression modules identified by WGCNA. Each leaf in the tree is one gene. The major tree branches constitute five modules labeled by different colors. **B** Module-sample relationship. **C** Module–bulbil number and -hormone contents relationships. The color bar represents the scale of the Spearman’s correlation. The color of the cell indicates the correlation coefficient between the traits: red indicates a high positive correlation and green indicates a high negative correlation. Asterisks indicate significant differences of these correlations detected by the Tukey’s HSD test (* *P* < 0.05 and ** *P* < 0.01) (B,C). **D-H** Heatmap is depicting the expression profile DEGs clustered in each module. **I**, Enrichment of KEGG pathway [34] (*P*-value < 0.05) annotation for five modular gene sets. The circle size of represents the counts of gene numbers involved in each KEGG pathway

A total of 1918 DEGs among US and LS samples were expressed with obvious selectivity systematically and clustered into five modules (green, blue, yellow, turquoise, and brown) by conducting WGCNA (Fig. 4A; Additional file 1: Fig. S9; Additional file 5: Data S3). Module brown was significantly correlated (0.85) with USs under decapitation, module blue showed a strong correlation with USs under IAA application (0.81 for HIUS and 0.43 for LIUS), and module turquoise was positively correlated with all LS samples (0.29–0.48) (Fig. 4B, D-H). We also analyzed the relationship between the module and some physiological traits. The changes in bulbil number between samples were correlated with module blue. The contents of IAA, ZR, and ABA were positively correlated with module blue, and ABA, GA, and JA were positively correlated with module brown. In contrast, the IAA, ZR, ABA, GA, JA were negatively correlated with module turquoise, and IAA and ABA were negatively correlated with module green (Fig. 4C). Results of KEGG annotation of each module showed that the removal or application of IAA dramatically affected gene expression connected to hormone-related pathways, including α-linolenic acid metabolism, phenylalanine metabolism, zeatin biosynthesis, phenylalanine, tyrosine and tryptophan biosynthesis, cysteine and methionine metabolism, terpenoid backbone biosynthesis, carotenoid biosynthesis, and plant hormone signal transduction (Fig. 4I).

**Fig. 5:**
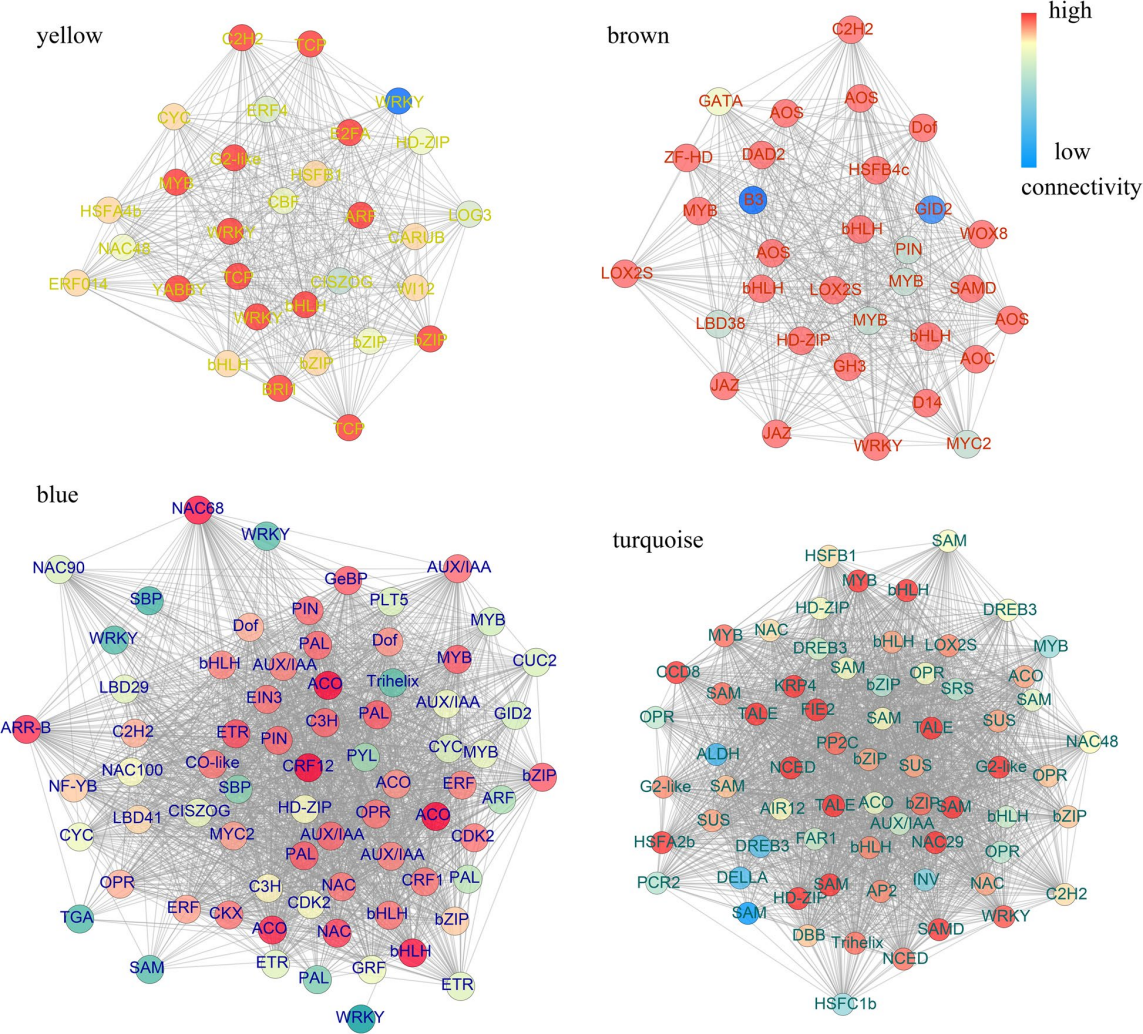
Network visualization of each co-expression module. Phytohormone and wound-related genes, and TFs are included in each module. The edges indicate connections between these genes. The node corlor ranged from red to blue representing the high to low connectivity

Researches have been made to confirm that the axillary meristem initiation is regulated by a set of transcription factors (TFs) [31, 32]. We identified 1516 TFs in the full-length lily transcriptome, referring to the TF amino acid sequences of Arabidopsis. The members of bHLH, GARS, C3H, WRKY, bZIP, and MYB families are in the majority (Additional file 1: Fig. S10). We identified total 209 candidate TFs that may function in bulbil outgrowth via a co-expression network. There were 73, 32, 7, 67, and 30 TFs identified in module blue, brown, green, turquoise, and yellow. To further investigate the main TFs related to bulbil induction, four networks (module yellow is excluded because TFs identified in this module are rare) were constructed and visualized using Cytoscape. As shown in Fig. 5, we manually selected hormone signaling genes, wound-induced genes, and the candidate TFs in four co-expression modules (Additional file 6: Data S4). Genes in module yellow were down-regulated at USs after treatments and the main TFs of this module contained two bHLHs (c4275/f1p1/5148 and c13970/f1p0/1107), two bZIPs (c4717/f3p1/1127, c7685/f2p1/1175, and c2614/f4p1/1069), three WRKYs (c13135/f2p1/1161, c13246/f1p0/2083, and c20523/f1p0/1199), three TCPs (c7013/f1p1/1011, c1123/f3p0/977 and c15895/f1p2/1044), one MYB (c8622/f3p0/1364) and one YABBY (c2167/f1p1/857) (Fig. 5). Module brown was significantly correlated with TUS sample, and TFs in this module were up-regulated after decaptation. We found three bHLHs (c11831/f1p2/1297, c11750/f1p2/1008, and c8598/f1p0/1504), three MYBs (c2197/f1p0/965, c1694/f1p0/984, and c12983/f1p0/2075), one C2H2 (c14477/f1p0/1170), and one WRKY (c4031/f1p0/5112) in module brown (Fig. 5). The co-expression network of blue module showing positive correlations with HIUS contained some other candidate TF genes, including three bHLHs (c19776/f1p0/1179, c12843/f1p0/1341 and c5184/f5p5/2273f), three MYBs (c13874/f1p0/1311, c24072/f5p6/1185 and c11157/f2p1/1083), five NACs (c11565/f1p0/2068, c20920/f1p0/1226, c2448/f1p1/988, c3262/f2p0/1113, and c4415/f1p0/962), two bZIPs (c6205/f1p0/1334 and c3433/f1p1/1171), and three WRKYs (c7658/f2p0/1144, c5479/f1p0/3291 and c5988/f1p0/1519) (Fig. 5). We also identified some TFs in module turquoise that may function in regulation of bulbil induction in LSs, including four bHLHs (c15312/f1p0/1748, c21093/f1p0/1578, c12826/f1p0/1062, and c13858/f1p0/1012), one C2H2 (c9288/f1p0/1210), four bZIPs (c9764/f1p0/1054, c21545/f1p6/1266, c1576/f9p3/1411, and c12838/f1p2/2125), three MYBs (c3676/f1p0/2077, c11562/f1p1/1688, and c15866/f1p0/1010), four NACs (c9275/f1p0/1204, c2434/f3p0/1219, c8344/f1p0/1004, and c15207/f1p1/1178), and one WRKY (c2926/f2p0/1071) (Fig. 5). The TFs that have high connectivity with wound- and hormone-related signals in the network may play key roles in bulbil induction. For example, in module blue, c19776/f1p0/1179, c6205/f1p0/1334, and c20920/f1p0/1226, that belonged to bHLH, bZIP, and NAC families showed 69, 44, and 66 degrees, indicating their potential in co-regulation of bulbil induction with wound- and hormone-related signals. Therefore, these TFs would be preference in further functional experiments.

## Discussion

Lily is an important bulbous plant with ornamental, medicinal, and edible uses [1]. Its giant genome (about 36 Gb) is expensive and challenging to the sequence. Previous large-scale sequencing of cDNA has been instrumental for gene discovery in lily, but the sequences rarely cover entire transcripts due to the limitation of NGS technologies [35, 36]. SMRT sequencing produces long reads and is particularly useful for non-model species lacking whole-genome sequencing data. Here, our results provide the first comprehensive, high-quality, full-length transcriptome of the lily cultivar ‘Sorbonne’ using a combination of SMRT-Seq and RNA-Seq, with correction of SMRT reads using Illumina reads. This accurate hybrid approach obtained 46,557 non-redundant transcripts from 39 equal mixed samples with N50 at 3452 bp. Moreover, the short reads produced by 39 RNA-Seq libraries were used for alignment against the PacBio datasets to calculate FPKM. Thus, all transcripts used for subsequent analysis are complete, reducing misassemblies of genes, especially in those gene families with high sequence identity. These data may greatly help researchers to study lily on the molecular level.

As aerial vegetative diaspores, the production of bulbils can act as an alternative or complement to sexual reproduction [2]. Bulbil formation is a rare natural phenomenon, but this character occurs in few *Lilium* species. Bulbils appear as small bulbs that have a short stem axis surrounded by layers of swollen scale leaves and are important vegetative reproductive organs in triploid *L. lancifolium*. Studies from *L. lancifolium* have showed that bulbil formation in *L. lancifolium* is a notable illustration of axillary organogenesis, by which a few parenchyma cells under the epidermis undergo dedifferentiation at the leaf axils and then differentiate into a bulbil structure [2, 4]. Previous transcriptome analysis has revealed that starch and sucrose metabolism and plant hormone signal transduction may play important roles in bulbil formation in *L. lancifolium* [4]. The cytokinin pathway promoted the initiation of bulbil formation, and iPA may an important type of cytokinin during bulbil formation in *L. lancifolium* [37]. Although some plants produce vegetative proliferations in response to environmental stress, mechanical injury, pathogens, or exogenous hormone treatment [2, 3, 5], it is still unclear in *Lilium* species without bulbils.

After decapitation or exogenous IAA application marked morphologic changes were observed, including differences in stem diameter, chlorophyll content, leaf width, and the size of bulblets. All morphologic changes are associated with nutrition accumulation and redistribution; accordingly, KEGG and GO annotations showed that DEGs were remarkably enriched in genes associated with carbohydrate metabolism, photosynthesis, and oxidation-reduction processes. Sugar has been reported to be necessary and sufficient to regulate the very earliest periods of bud outgrowth following decapitation [22]. Our study also showed that sucrose and starch metabolism genes were significantly altered after treatment. Furthermore, the most enriched genes were also associated with phytohormones, especially auxin. This finding is consistent with the formation of bulbils in triploid *L. lancifolium* and bulblets developed from scales in *L. davidii* var. *unicolor* [4, 11]. Based on these data, we proposed that auxin signals lead to morphologic changes and are essential for the subsequent development of aerial bulbils.

The induction of aerial bulbils at LSs can be explained as the release of apical dominance. In support of this hypothesis, auxin signals were significantly weakened after decapitation or LI treatment but enhanced after treatment with HI. Auxin depletion will differ spatially and temporally along the stem because auxin depletion is relatively slow, and therefore, the growing buds in the upper shoot will be affected before those lower on the stem. Auxin applied to the decapitated stump is unable to completely suppress decapitation-induced bud outgrowth in some species, such as *Phaseolus vulgaris* and *Arabidopsis* [38]. Strong auxin signaling completely repressed the outgrowth of bulbils at LSs. Accordingly, at LSs, the IAA concentration was still significantly elevated 12 hours after HI treatment, suggesting the repression of bulbil development is auxin dose-dependent. However, auxin can induce aerial bulbils around the cut surface at USs. Thus, the underlying mechanism of bulbil outgrowth at LSs or USs may be somewhat different.

An auxin-rich environment is essential for callus induction from explants, and high auxin promotes root development [39]. In this study, we observed callus-like cells close to the stem epidermis as well as many adventitious roots below the cut surface after treatment with HI but not after treatment with LI. Given all these data, including the fact that callus-like cells were induced at the cut surface after IAA application, we propose that the induction of aerial bulbils at USs occurs via a process similar to callus formation. Studies of de novo regeneration of bulblets have indicated that wounding activates a very fast regeneration response, which is responsible for triggering polar auxin redistribution, cell proliferation, and de-differentiation [40, 41]. Our RNA-seq data showed that wounding causes dramatic changes in gene expression profiles, and indeed many plant defense hormone signals displayed differential expression within 6 h after decapitation. Our analysis revealed a complex relationship between auxin treatment and the transcriptional responses of other hormone-related genes, implying that auxin can influence both of the cellular concentration and sensitivity to other plant hormones. There is strong evidence that other hormones are involved in crosstalk with auxin during axillary bud outgrowth. For example, the *AUX/IAA*-mediated BR signal has been observed in Arabidopsis [42]. Cytokinin is also known to impact polar auxin transport through the modulation of auxin efflux carrier activity [43]. In Arabidopsis roots, wounding of the tip rapidly changes the auxin distribution and creates a new auxin maximum, which results in de novo root meristem regeneration [44]. Therefore, we propose that in lily stems, wounding also causes an auxin transport disruption, which results in a transcriptional response involving genes, such as *ETR* and *TGA*, involved in plant defense [43, 45]. Wounding stimuli may induce regenerative responses via dynamic hormonal and transcriptional changes.

## Conclusions

In this report, we provide a fine assembly of transcriptome data which will pave the way for future research into lily function at the molecular level and will prove to be a rich resource for full-length genes that may be important for efforts aimed at improving or reducing bulbil number artificially. Wound stress and hormonal cues integrate and collectively regulate a large collection of genes implicated in bulbil developmental decisions at USs and LSs. Hormone signaling genes, wound-induced genes and some transcriptional factors that potentially regulate bulbil outgrowth were identified. Our work provides a crucial angle for an in-depth understanding of the molecular programs underlying lily’s unique bulbil induction processes.

## Materials and methods

### Plant materials

The Oriental hybrid ‘Sorbonne’ were grown in a greenhouse at the Institute of Botany, Chinese Academy of Science, Beijing, China (116°E, 40°N) under conditions of 25 °C (day)/20 °C (night) and daily sunlight. Two dormancy-breaking bulbs were propagated in a plastic pot (~ 25 cm in diameter) containing soil mixed with peat and sand at a ratio of 2:1. Plants were watered as needed and kept moist. When lilies were approximately 40 cm in height (noted as leaf-expansion developmental stages), plants samples including apex (CKA), upper stem (CKUS), lower stem (CKLS), leaf (CKL), prop root (CKPR), root (CKR), and bulbs (CKB) were harvested. Then the effects of auxin were examined by applying absorbent cotton containing lower concentration (30 mM, LI) or higher concentration IAA (300 mM, HI) (Beijing Lablead Biotech Co, Ltd) to the cut surface stump of decapitated plants. Upper stem and lower stem samples of topping plants (TUS, TLS) or plants treated with lower concentration IAA (LIUS, LILS) and higher concentration (HIUS, HILS) were collected after 12 h. All experimental solutions contained 0.5% Tween-20. All experiments were conducted in triplicate and 10 plants were pooled for each tissue. Harvested tissues were immediately frozen in liquid nitrogen and stored at − 80 °C.

### Hormone determination

Eight stem samples of different treatments and control plants were ground into powder using liquid nitrogen. The extraction and purification of endogenous hormones, including IAA, ZR, GA3, ABA and BR and were performed as previously [3]. Enzyme-linked immunosorbent assay (ELISA) was used for the estimation of hormone levels, with three biological replicates for each set of experiments.

### Observation of SEM

Three upper stem samples from three treatments were fixed in FAA fixation buffer (formaldehyde: glacial acetic acid: 70% ethanol, 1: 1: 18) at 4 °C. The fixed samples were dehydrated in a graded series of ethanol (70, 80, 90, 95, and 100%), followed by an ethanol/isoamyl acetate series (ethanol:isoamyl acetate 3:1, 1:1, and 1:3 and 0:1). Then the samples were dried using carbon critical point drying methods and prepared on metallic studs with double side tape. After ion coating with platinum by a sputter coater (JFC-1300, Jeol, Tokyo), morphology of stems was observed by SEM.

### RNA preparation

Total RNA was extracted from all finely grinded lily samples using a HiPure Plant RNA Mini Kit (Magen) according to the manufacturer’s instructions. The DNA was digested by DNaseI (Magen). The purity, concentration and integrity of RNA were assessed by the NanoPhotometer® spectrophotometer (IMPLEN, CA, USA), Qubit® RNA Assay Kit in Qubit®2.0 Flurometer (Life Technologies, CA, USA) and Agilent Bioanalyzer 2100 system (Agilent Technologies, CA, USA). Qualified RNA was stored at − 80 °C.

### Illumina short-read library construction and sequencing

Sequencing libraries were generated using NEBNext®Ultra™ RNA Library Prep Kit for Illumina® (NEB, USA) following manufacturer’s recommendations and index codes were added to attribute sequences to each sample. Total 3 μg RNA of each sample was enriched with oligo (dT)-rich magnetic beads and then broken into short fragments in Fragmentation Buffer. 1st strand cDNA synthesis was performed using random hexamers primer and M-MuLV Reverse Transcriptase (RNase H). 2nd strand cDNA was synthesized by adding reaction buffer, dNTPs, RNase H and DNA polymerase I. Next, the resulting cDNAs were subjected to end-repair, insert ‘A’ base and subsequently ligate with Illumina pairedend solexa adaptor. Adaptor-ligated fragments were purified by AMPure XP beads to select a size range of templates for downstream enrichment. Finally, PCR was performed to enrich and amplify the cDNA template. Twenty-one libraries of seven organs (apex, upper stem, lower stem, leaf, prop root, root, and bulbs) and eighteen libraries of upper and lower stem after three treatments were obtained with three repetitions for each sample. Sequencing was performed on an Illumina HiSeq™ 2000 at Biomarker Biotechnology Corporation (Beijing, China).

### PacBio long-read library construction and sequencing

Equal RNA from total of thirty-nine tissues was pooled into one RNA sample (5 μg) for PacBio single SMRT sequencing. Full-length (FL) cDNA was synthesized using SMARTer™ PCR cDNA Synthesis Kit. Amplification of the FL cDNA was followed by size fractionation (1–2, 2–3, 3–5, and 5–8 kb) using the BluePippin. The ends of FL cDNA were repaired and the hairpin sequencing adapters were ligated. Exonucleases were added to remove failed ligation products and screened by BluePippin again. Sequencing libraries were assessed for quality and quantity by Agilent 2100 and Qubit 2.0, respectively. The polymerase-bound SMRTbell libraries were placed onto the Pacific Bioscience RS II platform to sequence. We sequenced a total of 7 SMRT® Cells and the raw sequencing data are stored at the NCBI database.

### Error correction of SMRT-Seq consensus transcripts and transcriptome construction

Error correction for low-quality isoforms full-length polished consensus transcripts was performed with proovread software using an Illumina RNA-Seq data set consisting of more than 1385 million paired-end reads collected from above 39 libraries. Then we merged the error-correct low-quality and high-quality isoforms and reduce redundancy using CD-HIT (v4.6.8). The left files (read1 files) from all libraries/samples were pooled into one big left.fq file, and right files (read2 files) into one big right.fq file. Transcriptome assembly was accomplished based on the left.fq and right.fq using Trinity (v2.4.0) with min_kmer_cov set to 2 by default and all other parameters set default.

### Functional analysis

For functional annotation, we aligned the assembled unigenes to seven databases (NR, SwissProt, GO, COG, KOG, EggNOG, and KEGG) using BLALT (v2.11.0), KOBAS (v2.0), and HMMER (v3.0) software. The identification of CDS and SSR was conducted by TransDecoder and MIcroSAtellite identification tool (MISA, (http://pgrc.ipk-gatersleben.de/misa/misa.html) respectively. The wound- and hormone-related genes were identified by annotation of KEGG pathway [34]. The results of TF prediction and annotation based on gene sequence blast (e-value at 1e-10) to Arabidopsis TF database. Then the identity > 60% TFs were used for analysis.

### Differentially expressed genes analysis

The clean reads of RNA-Seq were aligned to full-length transcripts produced by SMRT sequence using Bowtie software (v2–2.1.0). The expression abundance of corresponding gene was represented by FPKM. The FPKM between the biological replications was analyzed using Pearson’s Correlation Coefficient (r) and the closer of r^2^ value to 1, the stronger correlation was between samples. The differentially expressed genes (DEGs) between various samples were identified and filtered with the R package DESeq2 (v1.36.0). We used FDR < 0.01 and the absolute value of log_2_(ratio) ≥ 2 as thresholds to define different gene expression. GO enrichment analysis of DEGs was implemented by the topGO R packages (v2.36.0) based Kolmogorov–Smirnov test. We used KOBAS software (v3.0) to test the statistical enrichment of differential expression genes in KEGG pathways.

### Gene co-expression network construction and visualization

A co-expression gene network was constructed using the WGCNA software package (v1.51) in R using all DEGs with FPKM≥0.1. Modules with default settings, except that the power is 8, minModuleSize is 20, and minimum height for merging modules is 0.33805. The networks were visualized using Cytoscape (v3.0.0).

## Supplementary Information

**Additional file 1: Figs. S1-S10.**

**Additional file 2: Tables S1-S3.**

**Additional file 3: Data S1.**

**Additional file 4: Data S2.**

**Additional file 5: Data S3.**

**Additional file 6: Data S4.**

**Additional file 7: Data S5.**

## Data Availability

The nucleotide sequence reported in this paper has been submitted to NCBI Sequence Reads Archive (SRA) with accession number PRJNA435633.
