# Supplementary material for: Full-length transcriptome-referenced analysis reveals crucial roles of hormone and wounding during induction of aerial bulbils in lily
Source: BMC Plant Biol. 2022 Aug 27;22:415. doi: 10.1186/s12870-022-03801-8 (PMC9419401; doi:10.1186/s12870-022-03801-8)
Supplement: Supplementary file 1 — Additional file 1: Figs. S1-S10. [file 12870_2022_3801_MOESM1_ESM.docx]

**Supplementary Figures**

**Title:** Full-length transcriptome-referenced analysis reveals crucial roles of hormone and wounding during induction of aerial bulbils in lily

**Authors:** Jingrui Li, Meiyu Sun, Hui Li, Zhengyi Ling, Di Wang, Jinzheng Zhang, Lei Shi^*^

**Additional file 1: Figure S1** Morphologic changes of leaf, LS and bulb after treatments. **Figure S2** Evaluation of SMRT-Seq data quality. **Figure S3** Comparison between Pacbio and Illumina. **Figure S4** Heatmap depicting the expression profile of differentially expressed genes. **Figure S5** Enrichment of KEGG pathway annotation for 1712 DEGs specific to US sample sets. **Figure** **S6** Enrichment of KEGG pathway annotation for 146 DEGs shared by US and LS sample sets. **Figure S7** Enrichment of KEGG pathway annotation for 60 DEGs specific to LS sample sets. **Figure S8** RT-PCR validations of expression patterns of DEGs related to auxin biosynthesis, transport and signal transduction. **Figure S9** Identification of gene co-expression modules by WGCNA. **Figure S10** The member number of each transcription factor family.


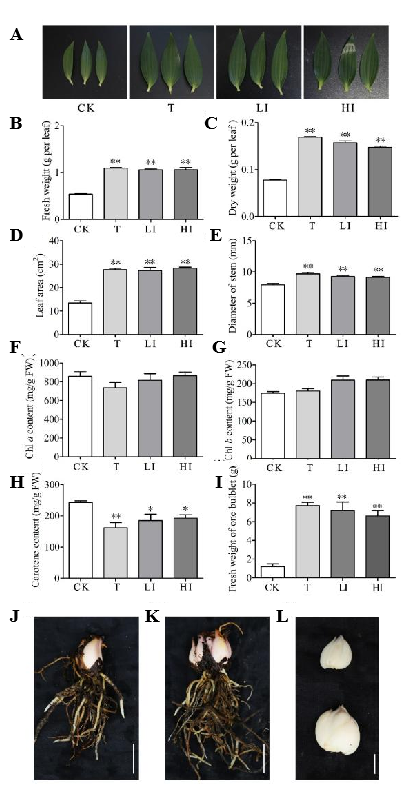


**Figure S1 Morphologic changes of leaf, LS and bulb after treatments.** (A) Leaves of control plants and treated plants. Two-week leaves were present after T, LI and HI at the leaf expansion stage in lily plants. (B-H) Comparison of physiological parameters for control plants versus treated plants. Fresh weight (B), dry weight (C), leaf area (D), stem diameter (E) and pigment contents (F–H) of leaf and fresh weight of one bulblet (I) were measured. Values represent the mean ± SE from 3 independent experiments. Asterisks indicate significant differences from control plants, as determined by the Tukey’s HSD test (*P<0.05, **P<0.01). (J-L) Bulbs of control plants (J) and decapitated plants (K). The bulblet of control plant was present at upper panel and that of decapitated plants was showed at lower panel (L). Scale bar = 5 cm (A,I,K); 2 cm (L).


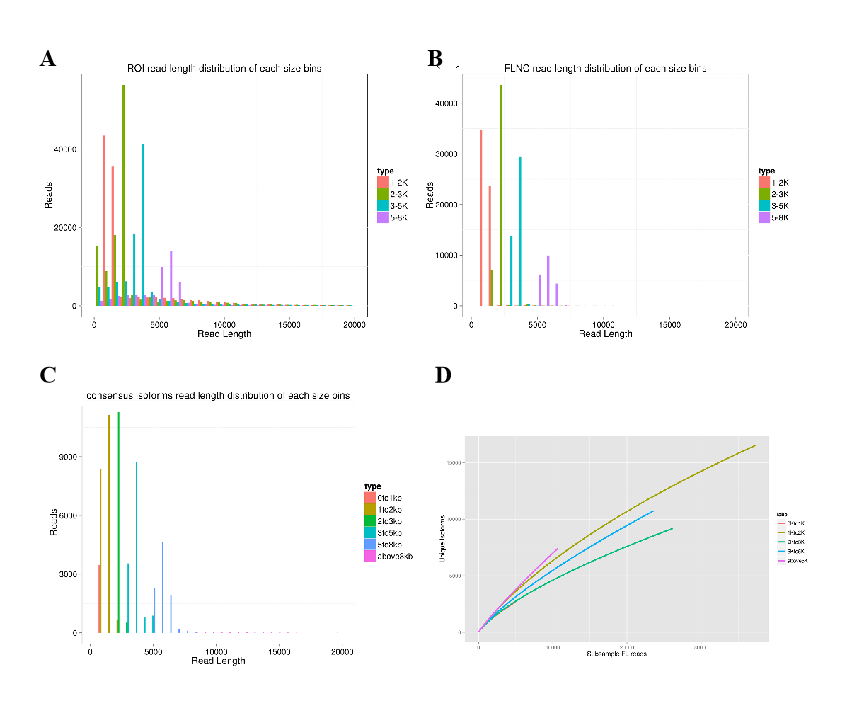


**Figure S2 Evaluation of SMRT-Seq data quality.** The ROI (A), FLNC (B) and consensus isoform (C) read length distribution and saturation analysis (D) of each size bins.

**
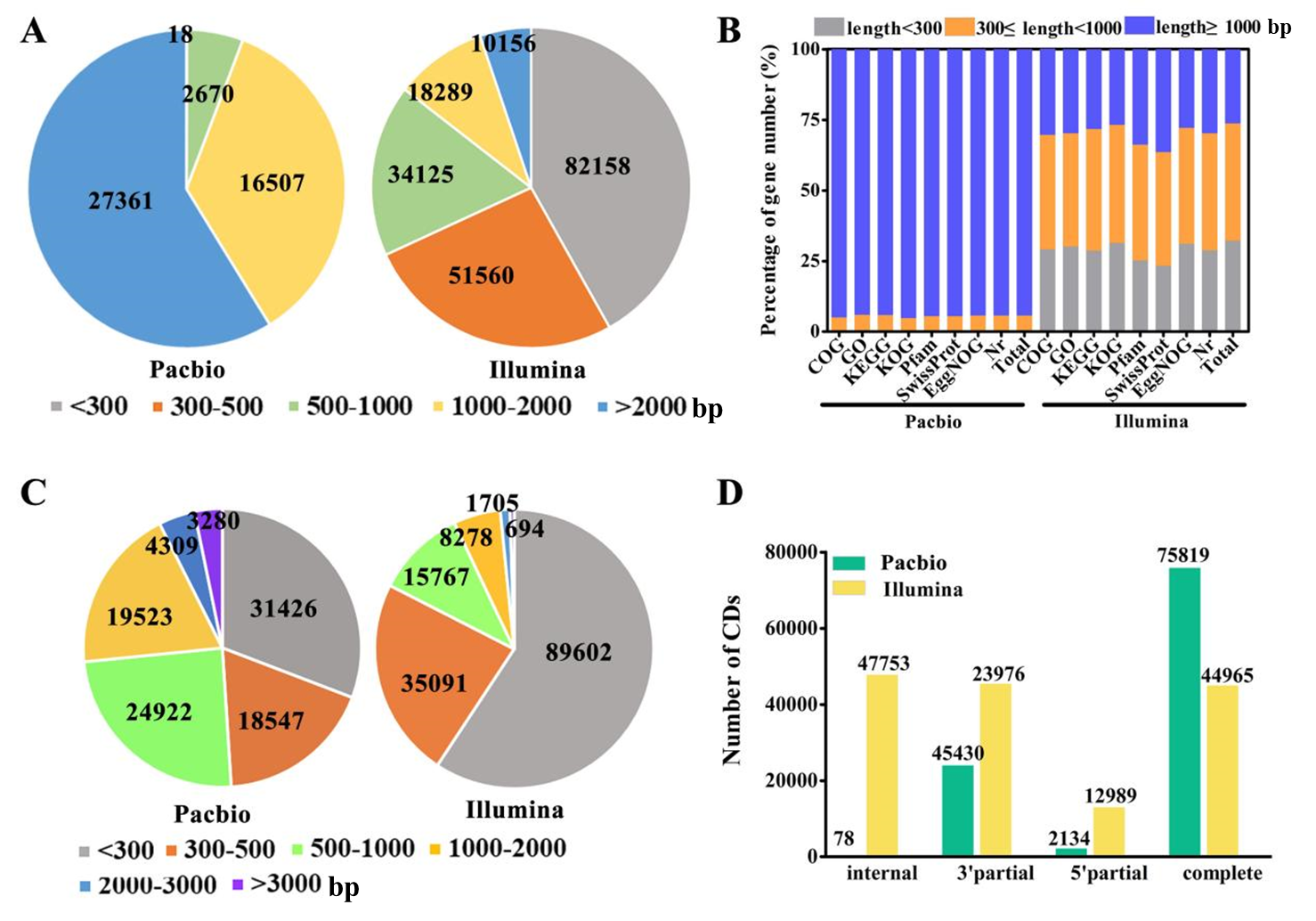
**

**Figure S3 Comparison between Pacbio and Illumina.** **A**, Pie chart showing the length distribution of transcripts from Pacbio and assembly unigenes from Illumina. **B**, Annotation of eight databases. **C**, Length distribution of CDS from Pacbio and Illumina. **D**, Distribution of internal CDS, CDS only with 5’-UTR or 3’-UTR, complete CDS in Pacbio and Illumina.


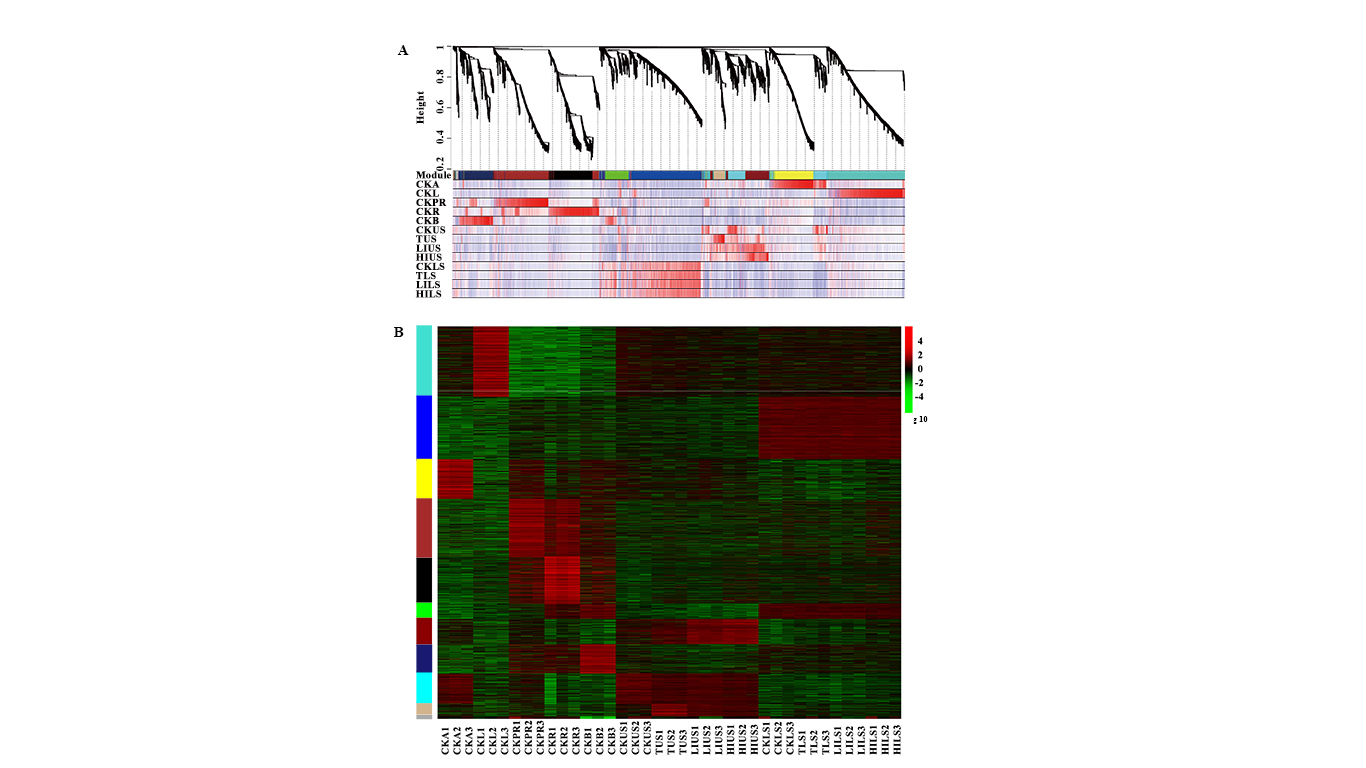


**Figure S4** **Heatmap depicting the expression profile of differentially expressed genes. A, The DEGs were clustered into 11 co-expression modules by WGCNA. B, Gene expression pattern in each module.**


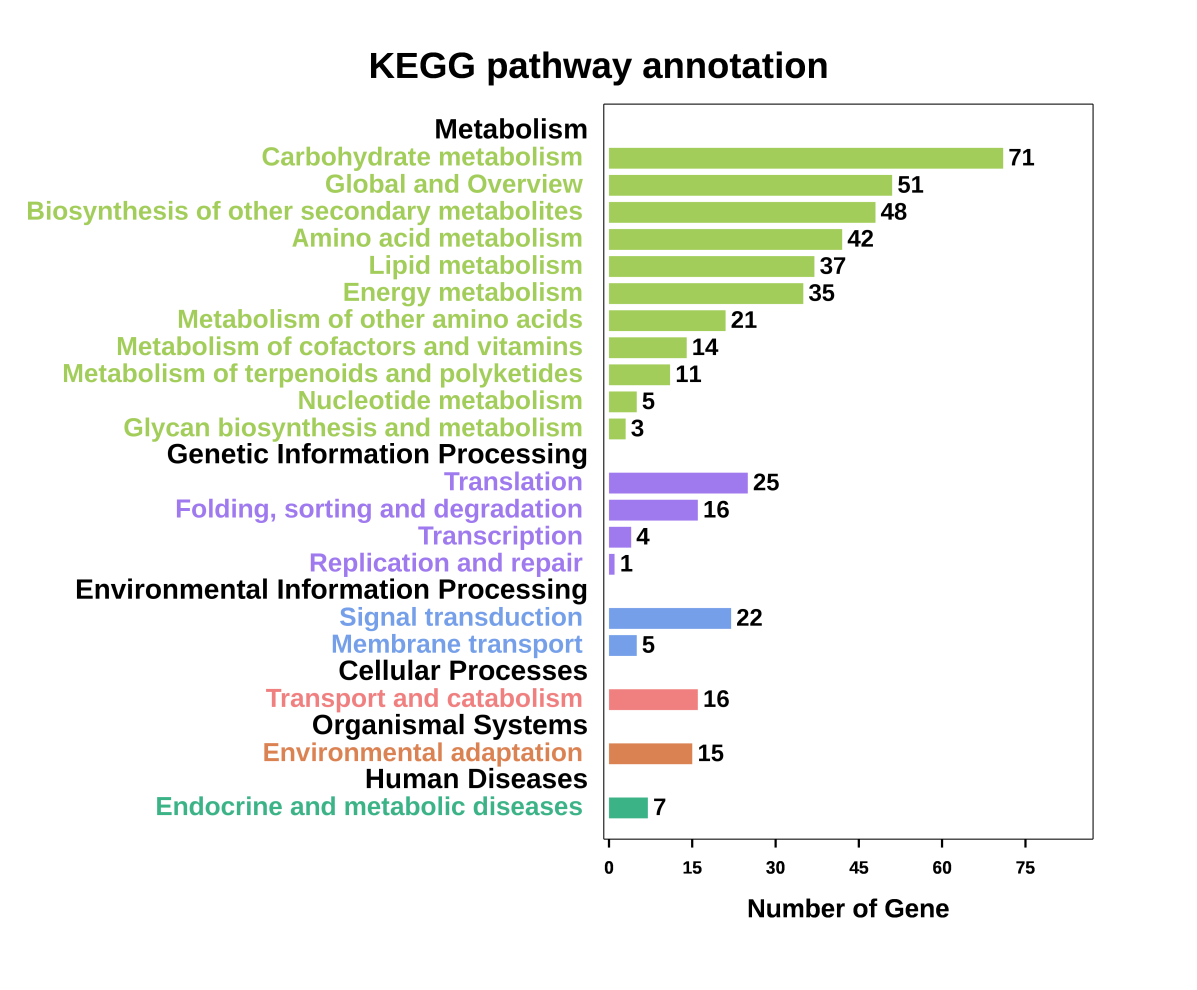


**Figure S5** Enrichment of KEGG pathway annotation for 1712 DEGs specific to US sample sets.


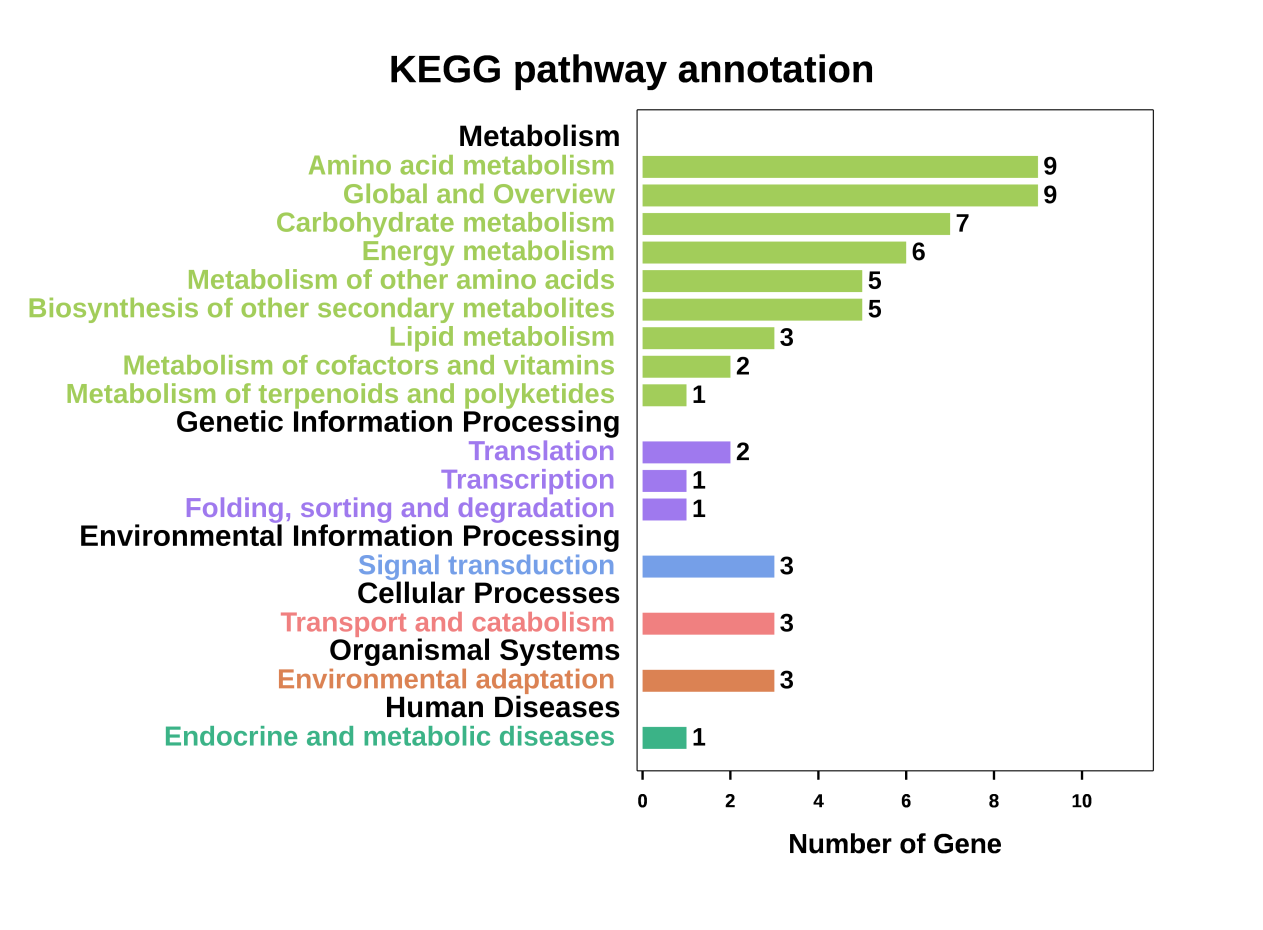


**Figure S6** Enrichment of KEGG pathway annotation for 146 DEGs shared by US and LS sample sets.


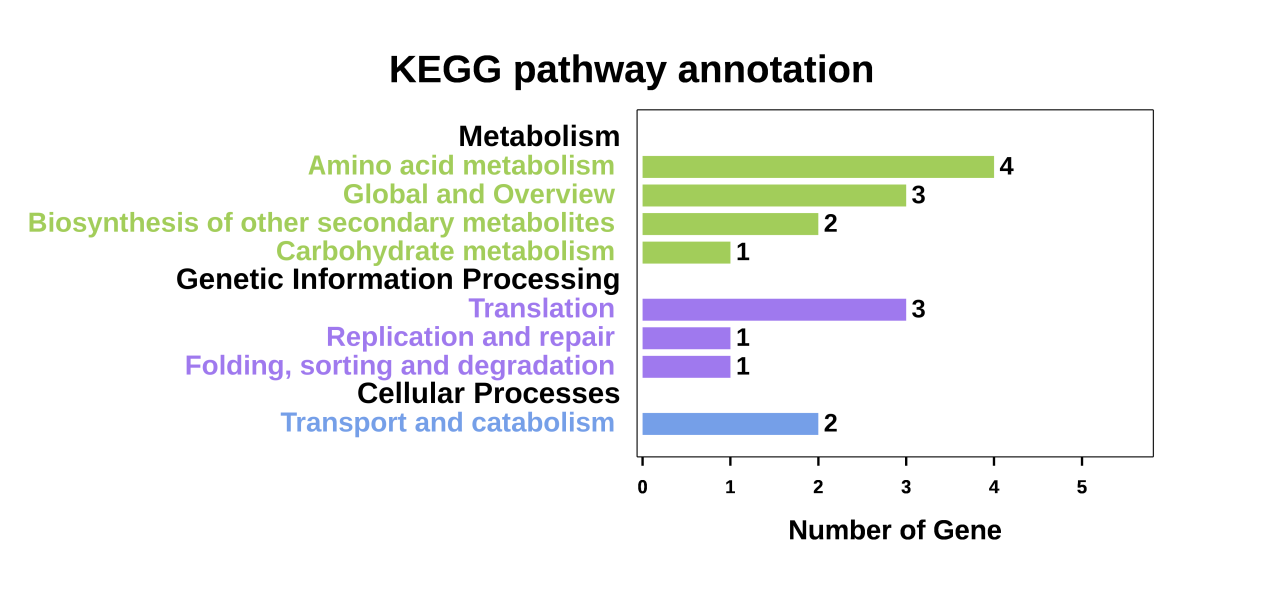


**Figure S7** Enrichment of KEGG pathway annotation for 60 DEGs specific to LS sample sets.


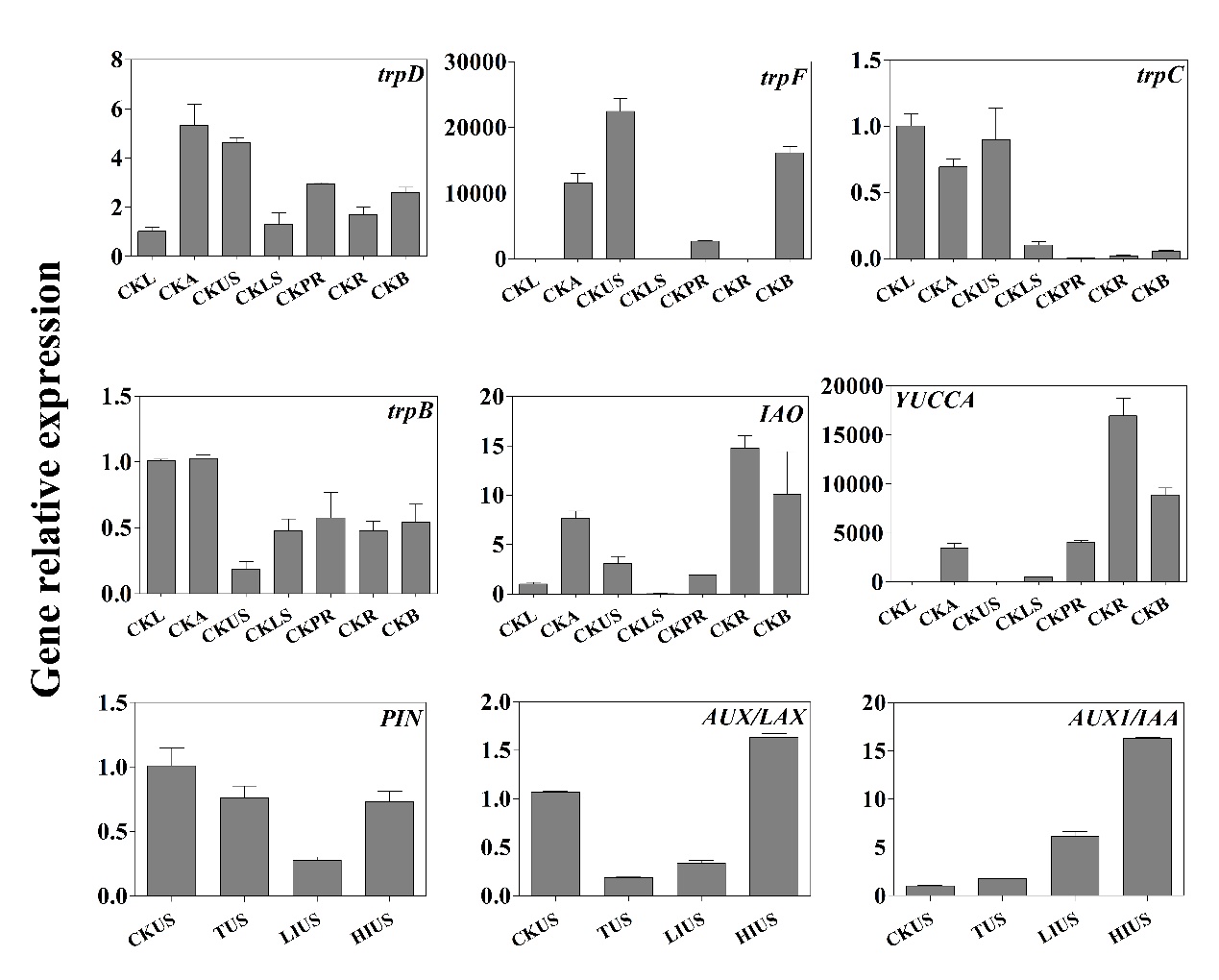


**Figure S8** qRT-PCR validations of expression patterns of DEGs related to auxin biosynthesis, transport and signal transduction. Bars represent the relative expression levels, normalized to that of *actin* transcripts. Values shown are mean ± SE of 3 replicates.


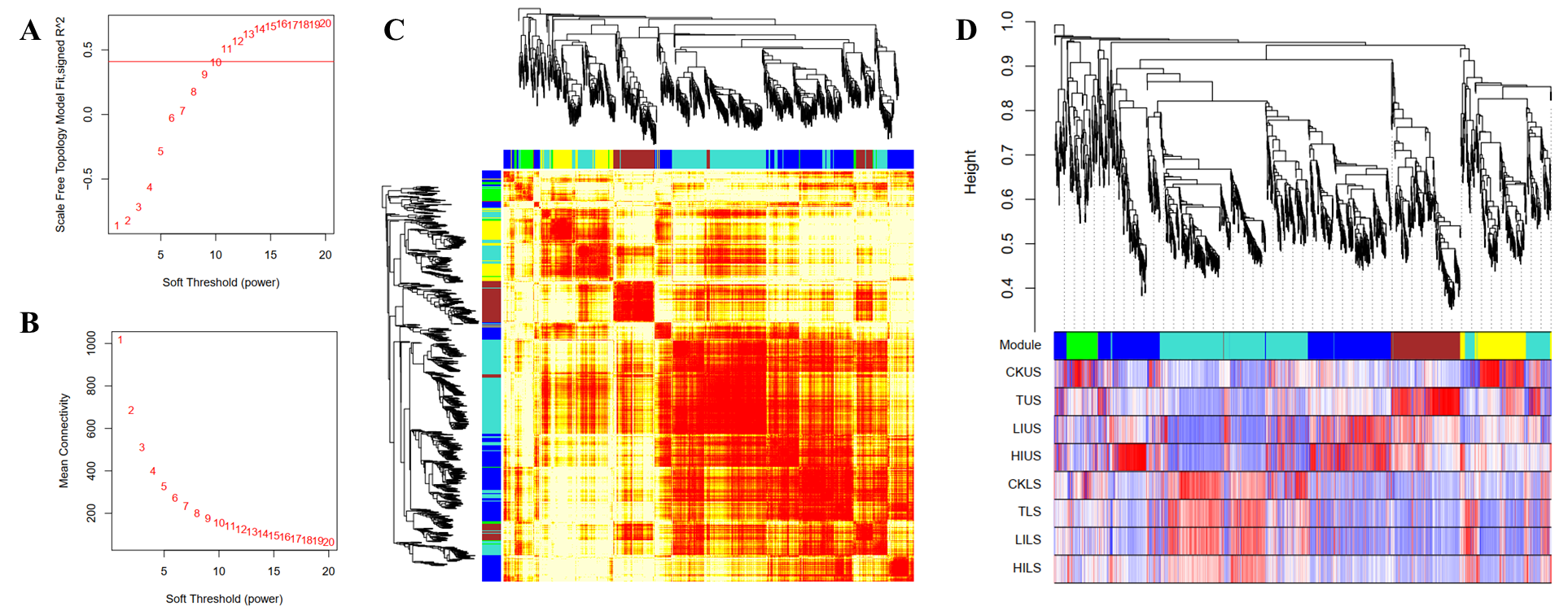


**Figure S9** Identification of gene co-expression modules by WGCNA. A,B Soft-Thresholding power is 10. C, Network heatmap plot for selected genes. D, The gene expression pattern of co-expression modules.

**
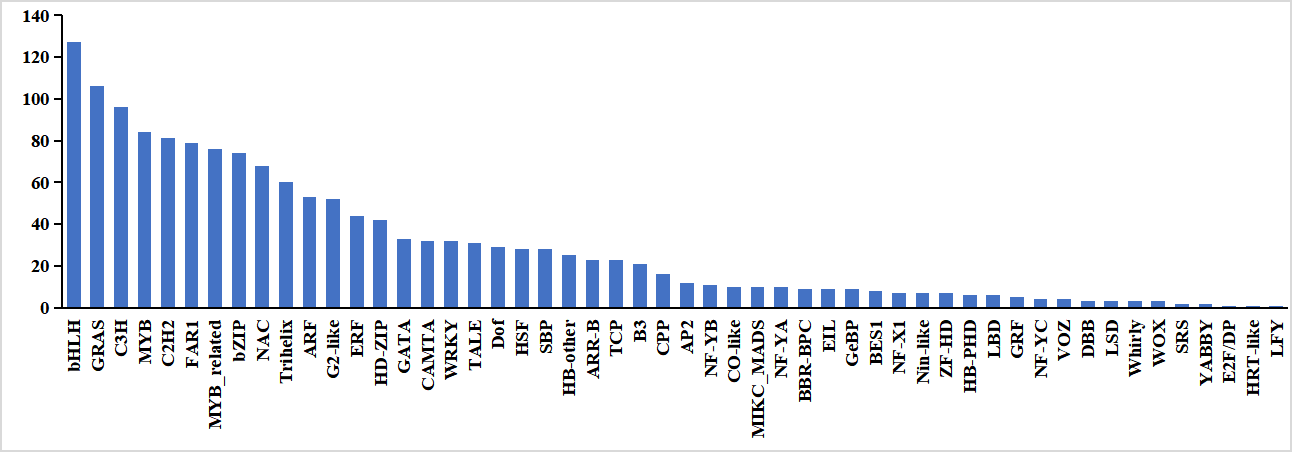
**

**Figure S10** The member number of each transcription factor family.
