# Supplementary material for: Full-length transcriptome-referenced analysis reveals crucial roles of hormone and wounding during induction of aerial bulbils in lily
Source: BMC Plant Biol. 2022 Aug 27;22:415. doi: 10.1186/s12870-022-03801-8 (PMC9419401; doi:10.1186/s12870-022-03801-8)
Supplement: Supplementary file 2 — Additional file 2: Tables S1-S3. [file 12870_2022_3801_MOESM2_ESM.docx]

**Supplementary Tables**

**Title:** Full-length transcriptome-referenced analysis reveals crucial roles of hormone and wounding during induction of aerial bulbils in lily

**Authors:** Jingrui Li, Meiyu Sun, Hui Li, Zhengyi Ling, Di Wang, Jinzheng Zhang, Lei Shi^*^

**Additional file 2: Table S1** Information of seven cells from SMRT sequencing. **Table S2** Information of Illumina reads from 39 libraries. **Table S3** Gene-specific primer pairs used for qRT-PCR.

**Table S1** Information of seven cells from SMRT sequencing.

| cDNA size | Cells | | Subreads | Reads of Insert | Number of five prime reads | Number of three prime reads | Number of poly-A reads | Number of filtered short reads | Number of non-full-length reads | Number of full-length reads | Full-length non-chimeric reads | Average full-length non-chimeric read length | Full-Length Percentage (FL%) | Artificial Concatemers (%) |
| --- | --- | --- | --- | --- | --- | --- | --- | --- | --- | --- | --- | --- | --- | --- |
| 1-2 K | 2 | 2814,893 | | 133,234 | 71,089 | 81,509 | 78,432 | 18,903 | 54,886 | 59,445 | 59,290 | 1,302 | 44.62% | 0.26% |
| 2-3 K | 2 | 1630,389 | | 121,213 | 64,390 | 71,289 | 69,782 | 10,451 | 59,046 | 51,716 | 51,681 | 2,187 | 42.67% | 0.07% |
| 3-5 K | 2 | 941,746 | | 96,919 | 53,157 | 58,742 | 58,183 | 2,946 | 50,075 | 43,898 | 43,597 | 3,422 | 45.29% | 0.69% |
| 5-8 K | 1 | 349,112 | | 50,509 | 26,790 | 29,408 | 29,141 | 588 | 29,220 | 20,701 | 20,662 | 5,551 | 40.98% | 0.19% |

**Table S2** Information of Illumina reads from 39 libraries.

| Samples | Clean reads | Base number | GC content | %≥Q30 | Mapped reads to Trinity assemble data | Mapped ratio to Trinity assemble data | Mapped reads to SMRT-seq data | Mapped ratratio to SMRT-seq data |
| --- | --- | --- | --- | --- | --- | --- | --- | --- |
| CKA1 | 33,705,078 | 9,962,436,370 | 50.74% | 92.55% | 22,080,075 | 65.51% | 19,668,092 | 58.35% |
| CKA2 | 33,682,796 | 9,961,235,198 | 50.49% | 92.71% | 22,493,448 | 66.78% | 20,059,013 | 59.55% |
| CKA3 | 35,577,487 | 10,487,621,758 | 50.85% | 92.62% | 23,853,720 | 67.05% | 21,370,108 | 60.06% |
| CKL1 | 35,355,146 | 10,440,235,334 | 51.66% | 92.75% | 23,170,925 | 65.54% | 21,215,895 | 60% |
| CKL2 | 42,607,855 | 12,587,481,434 | 52.01% | 92.61% | 29,337,708 | 68.86% | 26,928,836 | 63.20% |
| CKL3 | 50,398,145 | 14,914,601,386 | 52.15% | 92.80% | 34,054,345 | 67.57% | 31,575,777 | 62.65% |
| CKPR1 | 35,375,428 | 10,397,021,076 | 52.45% | 92.49% | 26,059,866 | 73.67% | 23,632,905 | 66.80% |
| CKPR2 | 46,570,978 | 13,780,683,578 | 51.57% | 92.25% | 33,016,902 | 70.90% | 29,872,185 | 64.14% |
| CKPR3 | 45,056,607 | 13,300,435,166 | 51.29% | 92.23% | 31,573,244 | 70.07% | 28,330,987 | 62.87% |
| CKR1 | 47,633,239 | 14,091,133,604 | 50.58% | 92.49% | 32,025,873 | 67.23% | 27,536,345 | 57.80% |
| CKR2 | 33,021,438 | 9,776,324,040 | 51.06% | 92.17% | 22,920,212 | 69.41% | 20,735,933 | 62.79% |
| CKR3 | 32,164,866 | 9,522,329,824 | 50.41% | 92.25% | 22,017,215 | 68.45% | 19,385,786 | 60.27% |
| CKB1 | 30,806,925 | 9,106,585,890 | 50.17% | 92.43% | 22,816,992 | 74.06% | 20,149,992 | 65.40% |
| CKB2 | 34,386,612 | 10,136,588,958 | 50.61% | 92.23% | 24,665,348 | 71.73% | 21,688,759 | 63.07% |
| CKB3 | 35,991,673 | 10,630,472,978 | 50.34% | 92.59% | 25,983,968 | 72.19% | 23,035,997 | 64% |
| CKUS1 | 39,628,964 | 11,746,891,674 | 50.83% | 92.64% | 26,628,062 | 67.19% | 24,144,482 | 60.92% |
| CKUS2 | 35,921,289 | 10,629,728,364 | 51.48% | 92.84% | 25,103,655 | 69.89% | 22,935,149 | 63.84% |
| CKUS3 | 36,541,697 | 10,797,023,788 | 51.82% | 92.88% | 27,104,791 | 74.17% | 24,799,093 | 67.86% |
| TUS1 | 30,525,112 | 9,071,151,924 | 51.11% | 93.36% | 24,820,032 | 81.31% | 22,141,237 | 72.53% |
| TUS2 | 30,737,937 | 9,152,395,870 | 51.19% | 93.21% | 24,994,169 | 81.31% | 22,390,809 | 72.84% |
| TUS3 | 34,043,436 | 10,063,562,266 | 51.32% | 93.75% | 27,762,258 | 81.55% | 24,867,262 | 73.04% |
| LIUS1 | 34,072,887 | 10,031,956,440 | 51.34% | 93.85% | 28,303,489 | 83.07% | 24,968,352 | 73.27% |
| LIUS2 | 31,102,553 | 9,228,018,872 | 50.71% | 93.70% | 25,853,456 | 83.12% | 22,683,067 | 72.92% |
| LIUS3 | 28,967,885 | 8,563,212,372 | 50.65% | 93.55% | 24,016,755 | 82.91% | 21,033,228 | 72.60% |
| HIUS1 | 32,923,607 | 9,701,268,914 | 51.43% | 93.05% | 27,288,329 | 82.88% | 23,936,334 | 72.70% |
| HIUS2 | 34,118,396 | 10,133,293,268 | 51.58% | 93.07% | 27,972,360 | 81.99% | 24,574,409 | 72.02% |
| HIUS3 | 31,943,324 | 9,465,601,458 | 51.52% | 93.17% | 26,446,821 | 82.79% | 23,327,565 | 73.02% |
| CKLS1 | 35,808,624 | 10,588,291,808 | 51.39% | 93.26% | 25,914,989 | 72.37% | 23,996,855 | 67.01% |
| CKLS2 | 30,280,507 | 8,940,162,690 | 50.49% | 93.18% | 21,596,835 | 71.32% | 19,828,264 | 65.48% |
| CKLS3 | 36,984,865 | 10,940,839,550 | 50.45% | 92.84% | 26,578,918 | 71.86% | 24,489,350 | 66.21% |
| TLS1 | 34,507,332 | 10,206,973,176 | 50.77% | 93.26% | 28,127,650 | 81.51% | 24,987,980 | 72.41% |
| TLS2 | 30,731,259 | 9,133,422,932 | 50.96% | 93.49% | 25,646,397 | 83.45% | 23,057,281 | 75.02% |
| TLS3 | 36,124,546 | 10,690,226,758 | 51.30% | 93.66% | 29,939,986 | 82.88% | 26,941,290 | 74.57% |
| LILS1 | 33,098,021 | 9,797,720,096 | 51.35% | 93.34% | 28,056,651 | 84.77% | 25,353,968 | 76.60% |
| LILS2 | 37,844,532 | 11,255,301,374 | 50.45% | 93.14% | 31,055,428 | 82.06% | 27,652,756 | 73.06% |
| LILS3 | 28,732,801 | 8,543,342,518 | 50.93% | 92.79% | 23,620,827 | 82.21% | 21,029,067 | 73.18% |
| HILS1 | 29,879,408 | 8,865,339,650 | 51.75% | 92.98% | 24,746,513 | 82.82% | 22,338,012 | 74.76% |
| HILS2 | 39,174,447 | 11,596,650,758 | 50.83% | 93.39% | 32,342,743 | 82.56% | 28,806,731 | 73.53% |
| HILS3 | 39,682,561 | 11,752,511,988 | 51.22% | 93.11% | 32,159,477 | 81.04% | 28,849,797 | 72.70% |

**Table S3** Gene-specific primer pairs used for qRT-PCR.

| Gene | Forward primer (5’-3’ sequence) | Reverse primer (5’-3’ sequence) |
| --- | --- | --- |
| *trpD* | ATCTCTACTGGGGCTTCGGT | CCAGCTTCCGTCACACATCT |
| *trpF* | CGAGGCTCTTGCTGCTCTTA | CAGACTCGATGCTTCGGCTA |
| *trpC* | GAACGAGGGAAACACTCCCC | ACGCAATACCCCTCGACTTG |
| *trpB* | CAAGCATAACCGATGCTGGC | TCAGAACGCTCCCCAAACAA |
| *IAO* | GACCACTACACACTCCTGCC | GTGGGCTGCACAAAGTTTCC |
| *YUCCA* | CCATCGTCGTTAGGAGTCCG | AACTGGATAAGCTGGGGTGC |
| *PIN* | GTTAACTCCTCAGGCCCACC | TATGCTAGTCCCGGCGGATA |
| *AUX/LAX* | ACCTATAGAAGCGCCAGTGC | CATGCTAGCCCATCCACCAA |
| *AUX/IAA* | TCGGTTAGTCGATTCGGCAG | ACTGCTCATAGCCCGCATAC |
| *actin* | CCATCCCAACCATGACACCA | TAAGCAATGGCTGATGCCGA |
